# Guidance for the collection of case report form variables to assess safety in clinical trials of vaccines in pregnancy^☆^

**DOI:** 10.1016/j.vaccine.2016.07.007

**Published:** 2016-12-01

**Authors:** Christine E. Jones, Flor M. Munoz, Sonali Kochhar, Stefania Vergnano, Clare L. Cutland, Mark Steinhoff, Steven Black, Ulrich Heininger, Jan Bonhoeffer, Paul T. Heath

**Affiliations:** a Paediatric Infectious Diseases Research Group, Institute of Infection and Immunity, St George’s, University of London, UK; b Baylor College of Medicine, Houston, TX, USA; c Global Healthcare Consulting, India; d School of Clinical Sciences, University of Bristol, UK; e Medical Research Council: Respiratory and Meningeal Pathogens Research Unit, South Africa; f Department of Science and Technology National Research Foundation, Vaccine Preventable Diseases, Faculty of Health Sciences, University of the Witwatersrand, South Africa; g Cincinnati Children’s Hospital Medical Center, USA; h Center for Global Health, Cincinnati Children’s Hospital, USA; i University of Basel Children’s Hospital, Basel, Switzerland

**Keywords:** Adverse event, Immunization, Pregnancy, Guidance, Clinical trials, Vaccines, Safety, Case report form

## Abstract

Vaccination in pregnancy is an effective strategy to prevent serious infections in mothers and their infants. Safety of this strategy is of principal importance to all stakeholders. As the number of studies assessing safety of vaccines in pregnancy increases, the need to ensure consistent collection and reporting of critical data to allow comparisons and data pooling becomes more important. The Global Alignment of Immunization Safety Assessment in Pregnancy (GAIA) project aims to improve data collection and create a shared understanding of maternal, fetal and neonatal outcomes in order to progress the global agenda for vaccination in pregnancy.

The guidance in this document has been developed to harmonize the data collected in case report forms used for safety monitoring in clinical trials of vaccination in pregnant women. Data to be collected is prioritized to allow applicability in diverse research settings, including low and middle-income countries. Standardized data will enable the research community to have a common base upon which to conduct meta-analyses, strengthening the applicability of outcomes to different settings.

## 1 Preamble

### 1.1 Background and need for this guidance

Vaccination in pregnancy is an effective strategy to prevent serious infections in mothers and their infants [1], [2], [3], [4], [5]. Recommendations exist for use of tetanus and influenza vaccines in many countries and the number of countries recommending pertussis vaccination continues to increase. Other vaccines are recommended in pregnant women where there is perceived benefit, such as hepatitis A, hepatitis B and meningococcal (serogroups A,C,W,Y). Novel vaccines targeting group B streptococcus (GBS) and respiratory syncytial virus (RSV) are in various stages of development [6], [7].

Safety of vaccination in pregnancy is a key consideration for pregnant women, healthcare providers, vaccine manufacturers, regulators, sponsors and ethics committees. The number of studies assessing safety of vaccination in pregnancy continues to increase; however, inter-study variability makes comparisons and pooling of data challenging [8]. The failure to collect and consistently report critical data and the absence of guidance for data collection were identified at two international conferences, which concluded that data collection and presentation should be harmonized across different studies and settings [9], [10].

The Global Alignment of Immunization Safety Assessment in Pregnancy (GAIA) project (http://gaia-consortium.net), coordinated by the Brighton Collaboration Foundation (https://brightoncollaboration.org), aims to improve data collection and create a shared understanding of maternal, fetal and neonatal outcomes in order to progress the global agenda for vaccination in pregnancy. The guidance proposed in this document have therefore been developed to harmonize the data collected in case report forms (CRFs) used for safety monitoring in clinical trials of vaccination in pregnant women. Guidance on the prioritization of the data to be collected is also provided to promote collection of at least a minimal set of high priority parameters in various settings, including low and middle-income countries (LMIC).

### 1.2 Use of this guidance

The aim of this guidance is to provide a standard for the collection of data in CRFs in clinical vaccine trials involving pregnant women where safety is an outcome. The guidance is presented as a series of tables and is referred to henceforth as a data collection matrix. It is intended as a tool to optimize data collection and to do so in a standardized manner in order to improve accuracy and comparability between clinical trials of vaccines in pregnancy. A standardized set of data will enable the research community to have a common base upon which to conduct meta-analyses, strengthening the applicability of outcomes to different settings. This data collection matrix is intended to be useful in all phases of clinical trials, from Phase I to Phase IV, from initial planning to implementation and evaluation. It is aimed at all stakeholders, from investigators, research networks, ethics committees and sponsors. It is also intended to be applicable in all resource settings; however, it is acknowledged that there are particular challenges to implementation in low and middle income countries (LMIC).

In consideration of this wide remit, data variables are prioritized into Priority 1 and Priority 2 data as follows:

*Priority 1:* Essential: data considered essential for the understanding of the trial results and/or required by national and/or international regulatory authorities
*Priority 2:* Complementary: data considered complementary, but not essential.

This data collection matrix is intended as a tool to assist all stakeholders; it is not regulatory or mandatory in nature. It is not intended to guide or establish criteria for clinical management. It is also not all-encompassing; it should be considered as a minimal data set in clinical trials where safety is an outcome. It is also expected that it may be adapted according to the specific aims and objectives of the individual clinical trial and that additional variables and data may also be collected.

It is intended that this guidance will be used alongside other existing guidelines from the Brighton Collaboration and the GAIA project. In particular, it is complementary to the “Guideline for collection, analysis and presentation of safety data in clinical trials of vaccines in pregnant women” [14]. The existing case definitions of key neonatal and maternal outcomes for clinical trials of vaccines in pregnancy, produced as part of GAIA should be referred to [14] as relevant and as new case definitions are developed these should also be used for safety assessments in future clinical trials. They will become available at www.brightoncollaboration.org.

### 1.3 Development process of data collection matrix

This data collection matrix was constructed using an iterative process. Six CRFs from investigator initiated and industry sponsored clinical trials carried out in diverse geographical settings, including Africa, Asia, Europe and North America, and assessing different vaccines were collected and all variables were extracted into Microsoft Excel (see acknowledgments section for contributors of the CRFs used). Each variable was then coded according to whether or not it was collected in each study. This enabled a visual representation of the variables in each study to be displayed. Each member of the Data Collection Matrix Working Group (CEJ, MS, PTH, SB, UH), then independently assessed each variable as essential, important or non-essential in clinical trials assessing safety of vaccination in pregnancy. Any other variables considered essential but missing from this master list were added at this stage. Each variable was then scored according to the number of individuals who considered it as essential or important. The list of variables for inclusion or exclusion was then reviewed and agreed by all members of the working group during a series of telephone conferences. Included variables were further refined during a process of review by the Executive Committee of the GAIA consortium by telephone calls and face to face meetings. Variables were grouped into tables and harmonized with the Guideline for collection, analysis and presentation of safety data in clinical trials of vaccines in pregnant women (referred to forthwith as the Guidelines document) [14]. The data collection matrix was then refined following structured peer-review by the broad global Brighton Collaboration Reference Group and review by subject matter experts attending the Harmonized Safety Monitoring of Immunization in Pregnancy International Consensus Conference and Investigators Workshop, 28–30th March 2016, National Institutes of Health, Bethesda, USA [11], [12]. This guidance should be considered as a ‘living document’, which will be reviewed periodically and updated to take account of emerging data and feedback from investigators implementing this guidance, these will be available at www.brightoncollaboration.org.

#### 1.3.1 Rationale for overall structure of the data collection matrix

The data collection matrix is presented as a series of tables of variables to be collected in case report forms. Each table relates to a different time-point or section of the case report forms.

### 1.4 Relationship of the data collection matrix to the “Guideline for collection, analysis and presentation of safety data in clinical trials of vaccines in pregnant women”

The data collection matrix and the Guidelines document are discrete documents, which are highly inter-related. It is expected that both documents will be used in parallel. The Guidelines document provides higher level information whereas the data collection matrix provides greater granularity. For example, the Guidelines document advises that safety follow-up should include a symptom diary to record solicited and unsolicited local and systemic adverse events following immunization (AEFI). The data collection matrix provides the detail of what variables should be collected in this symptom diary and suggests how signs and symptoms should be measured or graded. Each table in the data collection matrix relates to a section in the Guidelines document. Therefore, whilst distinct documents, they are harmonized and should be used together.

## 2 Guidance for the collection of case report form variables

The tables define data that should be collected prior to vaccination (Table 1), at the time of vaccination (Table 3) and in the follow-up period (Table 4). Early phase clinical trials may select pregnant women at low risk of obstetric complications, whereas in other studies, particularly late phase clinical trials, it may be preferable to enroll pregnant women regardless of their obstetric risk. In recognition of this, an example of variables that may be collected to assess obstetric risk is provided (Table 2).

In order to assess safety of vaccination in pregnancy, it is recommended that the minimum follow-up period for women is 6 months post-delivery or the early termination of pregnancy; the minimum recommended follow-up period for infants is until 1 year of age (Table 4). It is acknowledged that there are significant logistical challenges with extended follow-up periods; where a shorter follow-up period is pre-defined in the study protocol, adequate justification should be given, for example, based on biological characteristics of the vaccine, the vaccine-targeted disease or of the adverse event of specific interest, including patterns identified in previous trials. There may also be reasons for extending safety follow up further based on the above factors, or the characteristics of the vaccine recipient (e.g. nutrition, underlying diseases such as immune-depressing illnesses and other pre-existing conditions), or the intention to assess child development and late onset outcomes as part of the Risk Management Plan (which may require follow-up periods of 5 years or more).

Table 5 provides guidance for the collection of high quality data in order to allow interpretation of adverse events following immunization (AEFI); the purpose is not to provide guidance on assessment of a causal relationship. More detailed guidance on protocol development for clinical trials assessing vaccines is available elsewhere [13] (see Table 6).

## 3 Conclusions

This guidance is intended as a tool to optimize data collection in all phases of clinical trials of vaccination in pregnancy where safety is an outcome. The aim of standardizing data collection is to improve accuracy and comparability and to allow pooling of data between studies. The remit of this guidance is wide and therefore data variables are prioritized into essential data (priority 1) and data that are useful but less essential (priority 2). Variables collected in CRFs are expected to be dependent on the pre-specified aims and objectives, study setting and resources in the clinical trial. It is acknowledged that there are particular challenges of collecting all data variables in LMICs.

Evaluation of this tool will be the subject of future work in order to continue to improve the utility of the data collection matrix to all stakeholders in clinical trials of vaccines in pregnancy.

## Disclaimer

The findings, opinions and assertions contained in this consensus document are those of the authors. They do not necessarily represent the official positions or views of their respective institution or organization (e.g., government, university, or corporation).

## Figures and Tables

**Table 1:** Pre-vaccination screening data.

	Priority
*Maternal demographic details*	
Participant identifiers	1
Date of birth (dd/mm/yy or mm/yy, if actual date unknown) and age (completed years) of pregnant woman	1
Race	1
Ethnicity	1
Consanguinity (participant related to partner as second cousins or closer) (Y/N)	2
Highest maternal educational attainment (0–9)^a^	2
Household geographic location	1
Household environment (urban, suburban, rural)	2
Local indicators of socio-economic status^b^	2

*Study population*	
Inclusion criteria	1
Exclusion criteria	1
Date and time of informed consent	1
Details of person(s) giving consent (maternal participant, maternal participant’s partner/spouse, both)	2
Withdrawal of mother	1
Withdrawal of infant	1
Date of withdrawal	1
Reason for withdrawal	1
Loss to follow up (LTFU)	1
Date of LTFU	1
Reason for LTFU	1

*Maternal medical and obstetric history*	
Pre-existing medical conditions (diagnosis, diagnosis date, diagnosis stop date, or continuing medical condition)	1
Previous hospitalizations (with date of admission and discharge)	1
Previous inpatient surgical procedures (with date of admission and discharge)	1
If maternal HIV infection: WHO clinical HIV staging^c^	1
If maternal HIV infection: CD4 result (% and absolute), with date of test	1
If maternal HIV infection: Viral load test, with date	1
Concomitant medications (name of drug, route, start and stop date, reason for medication)	1
Medication history, including 1 month prior to pregnancy, to include prescription and non-prescription drugs, recreational drugs, herbal and homeopathic preparations and nutritional supplements	1
History of allergies, including allergen and reaction	1
Tobacco use in this pregnancy (e.g. daily, less than daily, never)^d^	1
Tobacco consumption in this pregnancy (e.g. average number per day or week/NA)	2
Alcohol consumption in this pregnancy (e.g. daily, several times a week or month, never)^e^	1
Gravity and Parity	1
Details of previous pregnancy complications (e.g. antepartum hemorrhage, post-partum hemorrhage, incompetent cervix)	1
Dates of previous deliveries, noting singleton or multiple births	1
Gestational age at delivery of previous infants	1
Type of previous delivery (vaginal, elective or unplanned cesarean section)	1
Outcome of previous pregnancies (live birth, still birth or abortion)	1
Previous neonatal deaths	1

*Current pregnancy*	
Date (dd/mm/yy) of last normal menstrual period (LMP)	1
Date of LMP: not known, unsure, sure	2
Estimated due date (EDD)	1
Method of EDD estimation (LMP, ultrasound, symphysis-fundal height or combination)	1
Dates of antenatal care visits in current pregnancy	1
Results of routine screening for infection	1
Results of routine screening to assess pregnancy and fetus (e.g. ultrasound, amniocentesis)	1
Vaccines received within 1 year prior to enrollment, with date of administration, where documentation available	1
Acute medical condition or pregnancy complication during current pregnancy	1
Results of non-routine laboratory testing during pregnancy relevant to current medical conditions	1
At high risk of serious obstetrical complications (Y/N) (example of obstetric risk assessment, Table 2)	2

*Maternal examination*	
Maternal weight (kg)	1
Maternal height (cm)	1
Maternal Body Mass Index, BMI, (or other validated nutritional indicator)	1
Vital signs, including: resting heart rate (bpm), Systolic and diastolic blood pressure (mmHg), Respiratory rate (breaths per minute), Body temperature (degrees Celsius or Fahrenheit)	1
General physical examination of mother (including general appearance, dermatological, cardiovascular, respiratory, hematological, gastrointestinal, urogenital, musculoskeletal, neurological, ocular/visual and endocrine/metabolic signs)	1
Obstetric examination (including scars from previous deliveries, fundal height, fetal heart tones and fetal movement)	1

*Maternal laboratory examinations*	
Date and results of general investigations (including Full blood count, differential, Urea, Creatinine, AST^∗^, ALT^∗^, GGT^∗^, Bilirubin, Na, K, Cl, Glucose [^∗^or equivalent])	1
Date and results of baseline investigations for infections that may impact on immunogenicity, efficacy and safety of pregnancy vaccines or aid interpretation of events that occur in the mother, fetus, neonate or infant	1
Date and results of investigations relevant to the target infection (e.g. GBS colonization in a GBS vaccine trial)	1
Date and results of urine tests (protein, glucose, bacterial culture)	1

*Fetal data*	
Presence of fetal growth restriction (IUGR)	1
Any fetal abnormality noted before vaccination of the mother by any screening test	1
Exposure to any other teratogens not noted above	1
Gestational age^f^ at time of vaccination of the mother	1

a Educational attainment levels should be categorized according to the International Standard Classification of Education (ISCED), a standard framework to ensure nationally comparable education statistics. 0 = Less than primary education, 1 = Primary education, 2 = Lower secondary education, 3 = Upper secondary education, 4 = Post secondary non-tertiary education (e.g. vocational training), 5 = Short cycle tertiary education (e.g. diploma of higher education), 6 = Bachelor’s degree or equivalent level, 7 = Master’s degree or equivalent level, 8 = Doctorate or equivalent level, 9 = Not elsewhere classified. Further guidance can be found here: http://www.uis.unesco.org/Education/Documents/isced-2011-en.pdf.

b Indicators of socio-economic status that are consistent between different settings are not easily defined. A multi-dimensional set of indicators including education, occupation, household income, assets and house status is ideal, but difficult to define for all settings. Indicators used by the UN Statistics Division can be found here: http://unstats.un.org/unsd/demographic/products/socind/.

c WHO case definitions of HIV for surveillance and revised clinical staging and immunological classification of HIV-related disease in adults and children. ISBN: 978 92 4 159562 9, 7th August 2006 http://www.who.int/hiv/pub/vct/hivstaging/en/.

d Further detailed guidance on questions assessing tobacco use can be found here: http://www.who.int/tobacco/surveillance/en/.

e Further detailed guidance on assessing alcohol consumption can be found here: http://www.niaaa.nih.gov/research/guidelines-and-resources/recommended-alcohol-questions.

f Guidance on the assessment of gestational age can be found in the GAIA case definition of preterm birth at brightoncollaboration.org [14].

**Table 2:** Example table of data that may be collected to inform obstetric risk assessment.

	Priority
*Maternal medical history*	
Diabetes mellitus, requiring medication (Y/N)	1
Hypertension requiring drug therapy (Y/N)^a^	1
Heart disease (Y/N)	1
Autoimmune disorder (Y/N)	1
Kidney disease (Y/N)	1
Neurologic disease (Y/N)	1
Psychiatric disorder requiring drug therapy (Y/N)	1
Hepatitis/liver disease (Y/N)	1
Varicosities/phlebitis of deep veins (Y/N)	1
Thyroid dysfunction (Y/N)	1
Pulmonary disease (Y/N)	1
Malaria in pregnancy (Y/N)	2
Substance abuse (Y/N)	1
D (Rh) sensitized (Y/N)	1
Three or more spontaneous abortions (Y/N)	1
Previous stillbirth^b^ or neonatal death (Y/N)^c^	1
Six or more previous deliveries (Y/N)	1
Previous infant with known genetic disorder (Y/N)	1
Previous infant with a major congenital anomaly (Y/N)^d^	1

*History of current pregnancy*	
Expected to deliver an infant at <37 weeks gestation (Y/N)	1
Expected to deliver multiple infants (Y/N)	1
Expected to deliver an infant with a major congenital anomaly (Y/N)^d^	1
Incompetent cervix (Y/N)	1
Polyhydramnios or oligohydramnios (Y/N)	1
Intrauterine growth retardation or fetal growth restriction (as above?) (Y/N)	1

a Case definition of gestational hypertension is available at brightoncollaboration.org [14].

b Case definition of stillbirth is available at brightoncollaboration.org [14].

c Case definition of neonatal death is available at brightoncollaboration.org [14].

d Case definition of congenital anomaly is available at brightoncollaboration.org [14].

**Table 3:** Vaccine and immunization related data.

	Priority
*Investigational vaccine and immunization procedures*	
Participant baseline vital signs (including blood pressure, heart rate, respiratory rate, and temperature)	1
Participant baseline symptoms assessment: temperature, headache, malaise, myalgia (none, mild, moderate, severe)	2
Vaccine allocation	1
Date & time of administration	1
Anatomical location of vaccine application	1
Administration route (e.g. oral, intramuscular, subcutaneous, intradermal, intranasal)	1
Actual dose volume administered	1
Lot number and expiry date of vaccine	1
Lot number and expiry date of any diluent	1
Name of person administering vaccine	1
Type of health care provider administering vaccine	2
Geographic location of vaccine administration	2
Temperature monitoring of vaccine (e.g. in storage, during transport)	2
Accountability of vaccine: documenting stocks	1
Post-vaccination medical observations (temperature [including anatomical site measured], heart rate, respiratory rate and blood pressure)	1
Post-vaccination symptoms assessment: temperature, headache, malaise, myalgia	2
Post-vaccination local reaction assessment	1
Details of simultaneous administration of other vaccines and indication	1

*Follow-up monitoring data (minimum 7–14 days post vaccination)*	
Daily temperature, including anatomical site of temperature monitoring	1
Systemic symptoms: chills, fatigue, malaise, myalgia, headache, arthralgia, nausea, rash (none, mild, moderate or severe)	1
Local vaccination site reactions: pain (none, mild, moderate or severe) erythema, induration, swelling or bruising (all in mm), itching (none, mild, moderate or severe)	1
Non-solicited symptoms: Y/N, if Y: description and severity (mild, moderate or severe)	1
Need for analgesia or antipyretic	2

**Table 4:** Follow-up monitoring data (including data pertaining to the mother pre-delivery, fetus, the delivery and the neonate, mother and infant until completion of follow-up).

	Priority
*Maternal (pre-delivery) & fetal follow-up*	
Date of visit	1
Location of study visit (telephone, clinic visit, home visit)	2
Gestation at study visit	1
Details of new medical conditions, surgical procedures or hospitalizations	1
Details of any symptoms or signs of infection vaccinated against in study	1
Details of any changes to previously reported adverse event	1
Details of any additional pregnancy monitoring (e.g. ultrasound, amniocentesis)	1
Results of any additional antenatal screening of infection	1
Results of any additional laboratory testing	1
Details of any new medications or changes to existing medications (name of drug, route, start and stop date, reason for medication)	1
Details of any further vaccines delivered (including date of administration)	1
Details of any protocol deviations	1
Vital signs, including: resting heart rate (bpm), Systolic and diastolic blood pressure (mmHg), Respiratory rate (breaths per minute), Body temperature (degrees Celsius or Fahrenheit)	2
General physical examination of mother (including general appearance, dermatological, cardiovascular, respiratory, hematological, gastrointestinal, urogenital, musculoskeletal, neurological, ocular/visual and endocrine/metabolic signs)	2
Obstetric examination (including scars from previous deliveries, fundal height, fetal heart tones and fetal movement)	2
Maternal BMI	1
If study visit missed, reason for study visit not occurring	1

*Delivery details*	
Date and dose of betamethasone administered during pregnancy, where relevant	2
Start and end date of any antibiotics received in last 1 week, specifying antibiotic received	2
Date and time of each dose of antibiotic in labor	1
Maximum temperature recorded during labor (degrees Celsius or Fahrenheit, with date and time)	2
Geographic location of delivery	1
Setting of delivery (home, clinic, hospital)	1
Delivery center admission and discharge dates	2
Date and time of delivery (include for each infant delivered if multiple infants)	1
Delivery mode: vaginal, C-section (elective, semi-elective, emergency)	1
Details of health care assistant present at delivery (none, midwife, physician, other)	2
Length of first stage and second stage of labor	2
Evidence of non-reassuring fetal status^a^ (Y/N, specify evidence)	2
Date and time of rupture of membranes	2
Details of decreased level of amniotic fluid	2
Meconium staining of amniotic fluid	2
Suspicion of chorioamnionitis: Y/N and specifying parameters used to assess (maternal fever, tachycardia, uterine tenderness, fetal tachycardia)	2
Details of any new antenatal complications e.g. pre-eclampsia,^b^ eclampsia	2
Details of any delivery complications e.g. induction/convulsions/lacerations or episiotomy	2
Requirement for general anesthetic during delivery	2
Results of any peri-natal laboratory tests pertinent to assess maternal safety	1
Post partum complications (e.g. fever, endometritis, wound infection, retained placenta, post-partum haemorrhage^c^)	1

*Infant details at birth*	
Number of infants born	1
Birth related vitality status of each infant: live birth, stillbirth,^d^ neonatal death^e^	1
If stillbirth,^d^ gestational age at birth (weeks and days)	1
Infant gestational age^f^	1
Method of assessment of infant gestational age (e.g. by estimated due date, by examination of external physical characteristics)	1
Infant birth weight (g)	1
Infant birth length (cm)	1
Infant birth head circumference (cm)	1
Infant gestational size (e.g. SGA	1
Infant sex (female, male, indeterminate)	1
Infant ethnicity/race	1
Infant APGAR score (or other immediate assessment for signs of life) at 1, 5 and 10 min	1
Need for infant resuscitation with details	1
Specify any abnormalities on newborn examination	1
Infant feeding: breast milk (mother/donor), formula feeding, parenteral nutrition, mixed feeding, other (specify nutrient given), with start dates	2
Results of any neonatal laboratory tests specified in the protocol (minimum of full blood count, differential, transaminases, bilirubin, glucose, blood urea nitrogen and creatinine)	1

*Neonatal complications*	
Admission to neonatal unit: none, transitional care, special care, intensive care	1
Dates of admission and discharge to neonatal unit	1
Respiratory abnormalities	1
Respiratory support required: Y/N, details	1
Dermatological abnormalities	1
Cardiovascular abnormalities	1
Hematological abnormalities	1
Gastrointestinal abnormalities	1
Neurological abnormalities (including results of audiological testing)	1
Musculo-skeletal abnormalities	1
Urogenital abnormalities	1
Ocular/visual abnormalities	1
Endocrine/metabolic abnormalities	1
Neurodevelopmental abnormalities	1
Signs of congenital malformations^g^ or birth injuries	1
Signs of congenital or acute infection^h^ in neonate	1
Neonatal death^e^(Y/N, details)	1
Where relevant, autopsy/verbal autopsy details	1
Details of any medical/surgical treatment received	1
Where relevant, infant hospital discharge: date/time	1

*Infant follow-up visits (minimum follow-up of 1 year recommended)*	
Date of visit	1
Age at visit	1
Location of study visit (phone, clinic, home visit)	1
Source of information (e.g. mother, father, other family member, carer, medical records)	1
If visit not occurred, reason why visit missed	1
Details of new medical conditions with diagnosis and date of diagnosis	1
Surgical procedures with dates	1
Details of medical reviews with dates	1
Hospitalization (Y/N), additional details below	1
New medical diagnoses with date of diagnosis	1
Details of any symptoms or signs related to infection that vaccine intended to prevent	1
Details of any changes to previously reported adverse event	1
Details of any new medications (prescribed and non-prescribed) or changes to existing medications (name of drug, route, start and stop date, reason for medication)	1
Infant vaccination details including date of administration	1
Infant feeding modality: breast/replacement/mixed (specify type of nutrient given) with start and stop dates	2
Infant weight (g)	1
Infant length (cm)	1
Infant head circumference (cm)	1
Infant medical examination	1
Infant developmental assessment	1
HIV-exposed infants: PCR test result, date	1

*Maternal follow-up visits (minimum follow-up of 6 months post delivery)*	
Date of study visit	1
Time interval since delivery (e.g. days, weeks)	1
Location of study visit (telephone, clinic, home)	1
If visit not occurred, reason why visit missed	1
Details of new medical conditions, surgical procedures, or hospitalizations	1
Details of any symptoms or signs of infection vaccinated against in study	1
Results of any laboratory tests with dates	1
Details of any changes to previously reported adverse event	1
Details of any new medications or changes to existing medications (name of drug, route, start and stop date, reason for medication)	1
Details of any vaccines delivered since study vaccine (including date of administration)	1
Details of any protocol deviations	1
Vital signs, including: resting heart rate (bpm), Systolic and diastolic blood pressure (mmHg), Respiratory rate (breaths per minute), Body temperature (degrees Celsius or Fahrenheit)	2
Maternal BMI	1
General physical examination (including general appearance, dermatological, cardiovascular, respiratory, hematological, gastrointestinal, urogenital, musculoskeletal, neurological, ocular/visual and endocrine/metabolic signs)	2

*Details of maternal or infant hospital admission*	
Date of admission and discharge	1
Admission location	1
Intensity of care: ward/high dependency/intensive care	1
Date of onset of symptoms	1
Symptom details	1
Antibiotics received prior to admission	1
Admission medical observations	2
If infant, infant admission weight	1
Admission due to obstetric complication: Y/N	1
Results of laboratory tests with dates	1
Results of radiology tests with dates	1
Diagnosis	1
Treatment received	1
Discharge outcome: discharged without sequelae, discharged with sequelae, still in hospital, death, AE number relating to hospitalization	1

a Case definition of non-reassuring fetal status is available at brightoncollaboration.org [14].

b Case definition of gestational hypertension is available at brightoncollaboration.org [14].

c Case definition of postpartum haemorrhage is available at brightoncollaboration.org [14].

d Case definition of stillbirth is available at brightoncollaboration.org [14].

e Case definition of neonatal death is available at brightoncollaboration.org [14].

f Guidance on the assessment of gestational age can be found in the GAIA case definition of preterm birth at brightoncollaboration.org [14].

g Case definition of congenital anomaly is available at brightoncollaboration.org [14].

h Case definition of neonatal infection is available at brightoncollaboration.org [14].

**Table 5:** Adverse event monitoring data (including maternal, fetal and infant).

	Priority
*Adverse event following immunization (AEFI) data collection*	
Participant number	1
Adverse event number	1
Name of person reporting AEFI	1
Contact details of reporter	1
Source of information (e.g. hospital, clinic notes)	1
Role of reporter (e.g. physician, nurse, midwife)	1
Date and time of report	1
Modality to capture event (e.g. scheduled trial visitor phone call, unscheduled visit)	1
Study vaccine allocation	1
Administration route of study vaccine (e.g. oral, intramuscular, subcutaneous, intradermal, intranasal)	1
Date of study vaccination	1
Date and time of first onset/observation & temporal relationship to vaccination	1
Detailed history of event(s), including times/dates	1
Initial diagnosis	1
Date of diagnosis	1
Type of AEFI with details (e.g. dermatological, cardiovascular, hematological, gastrointestinal, neurological [includes audiological], respiratory, musculoskeletal, urogenital, ocular/visual, endocrine/metabolic,	1
If infant: Small for gestational age (Y/N)	1
If infant: details of any congenital anomalies^a^ or birth injuries	1
If infant: congenital infection (Y/N, details)	1
If pregnant woman include details of fetal distress, time of miscarriage or still birth	1
Participant seen by physician for complaint: Y/N/U	1
Contact information of physician	2
Hospitalization for current event (Y/N/U) with dates of hospitalization	1
Contact details of hospital	2
Admission date of hospitalization	1
Concomitant symptoms, sign and diseases (other than those described)	1
Findings from physical examination	1
Findings from further investigations (test, date, findings, diagnosis, source of information)	1
Treatment for AEFI (dates, clinical condition and treatment provider)	1
History of recurrence of the event after AEFI	1
Outcome of event/action taken	1
Severity of AEFI (Hospitalization/disability/life threatening event/death [with details of cause and autopsy details])	1

*AEFI follow-up data collection*	
Participant number	1
Adverse event number	1
Name of person reporting AEFI	1
Contact details of reporter	1
Role of reporter – e.g. investigator	1
Date and time of report	1
Final diagnosis	1
Date of final diagnosis	1
Causality assessment done: Y/N/U	1
Outcome of causality assessment^b^: not related/related/indeterminate (definitions and justification should be documented in study documentation)	1
Alternative etiology: Y/N, details	1
Participants condition return to pre-vaccination health status: Y/N/U	1
Resolved (Y/N) and date of resolution	1
Criteria for SAE reached: Y/N/U	1
Outcome of AEFI	1
If died: date of maternal^c^ or neonatal death^d^	1
Cause of death	1
Autopsy performed Y/N, outcome	1
Further doses of investigational product given: Y/N and dates	1
Details of any concurrent local disease outbreaks pertinent to AEFI	1

a Case definition of congenital anomaly is available at brightoncollaboration.org [14].

b Guidance of causality assessment can be found at: https://brightoncollaboration.org/public/resources/standards/guidelines.html.

c Case definition of maternal death is available at brightoncollaboration.org [14].

d Case definition of neonatal death is available at brightoncollaboration.org [14].

**Table 6:** Protocol deviations and additional data.

	Priority
*Protocol deviation*	
Protocol deviation date	1
Protocol deviation description	1
Reason for protocol deviation	1
Effects of protocol deviation: AE/termination of FU/product stability	1

*Other*	
Emergency unblinding Y/N	1
Emergency unblinding reason	1
Unscheduled visit Y/N	1
Reason for unscheduled visit	1
Sample storage	1
Blood sampling details: date, time, volume	1
